# The causal relationship between type 2 diabetes mellitus and isolated REM sleep behavior disorder: results from multivariable and network Mendelian randomization analysis

**DOI:** 10.3389/fendo.2024.1408053

**Published:** 2024-11-25

**Authors:** Ru-Yu Zhang, Jin-Yu Li, Yu-Ning Liu, Zi-Xuan Zhang, Jie Zhao, Fu-Jia Li

**Affiliations:** ^1^ Department of Pulmonary and Critical Care Medicine, First People's Hospital of Zigong, Zigong, Sichuan, China; ^2^ Department of Respiratory and Critical Care Medicine, The Second Affiliated Hospital of Xuzhou Medical University, Xuzhou, Jiangsu, China; ^3^ Department of Neurology, The Affiliated Hospital of Xuzhou Medical University, Xuzhou, Jiangsu, China; ^4^ Department of Neurology, The Eighth Affiliated Hospital, Sun Yat-Sen University, Shenzhen, Guangdong, China

**Keywords:** type 2 diabetes mellitus, isolated REM sleep behavior disorder, Mendelian randomization, genetic correlation, causal association

## Abstract

**Objectives:**

To investigate the causal relationship between type 2 diabetes mellitus (T2DM, exposure) and isolated REM sleep behavior disorder (iRBD, outcome).

**Methods:**

Genome-wide association study (GWAS) data for iRBD comprised 9,447 samples, including 1,061 iRBD cases from the International RBD Study Group. Initially, we performed linkage disequilibrium score regression (LDSC) to explore the genetic correlation between T2DM and iRBD. Then the two-sample univariate MR (UVMR) analysis was conducted to examine the effects of T2DM and blood sugar metabolism-related factors on iRBD. Subsequently, we applied multivariable MR (MVMR) methods to further adjust for confounders. Lastly, we executed a network MR analysis, with cytokines and immune cell characteristics as potential mediators, aiming to investigate indirect effect of T2DM on iRBD.

**Results:**

Results from LDSC suggest a genetic correlation between T2DM and iRBD (rg=0.306, P=0.029). UVMR analysis indicates that both T2DM (Odds Ratio [95% Confidence Interval] = 1.19 [1.03, 1.37], P = 0.017) and high blood glucose levels (1.55 [1.04, 2.30], P = 0.032) are risk factors for iRBD. Even after adjusting for confounders in MVMR, the association between T2DM and iRBD remains robust. Finally, results from network MR analysis suggest that T2DM may indirectly promote the development of iRBD by reducing levels of Stromal Cell-Derived Factor 2 in circulation and by increasing BAFF-receptor expression in IgD- CD38- B cells.

**Conclusions:**

T2DM may promote the onset of iRBD by influencing immune-inflammatory responses. Our findings provide valuable insights and directions for understanding the pathogenesis of iRBD, identifying high-risk groups, and discovering new therapeutic targets.

## 1 Introduction

Isolated rapid eye movement (REM) sleep behavior disorder (iRBD), a parasomnia characterized by the lack of muscle atonia and abnormal behaviors during REM sleep, significantly impacts sleep quality and poses safety and health risks to both patients and their bed partners (1). Critically, iRBD serves as a known precursor for severe neurodegenerative α-synucleinopathies such as Parkinson’s disease (PD), multiple system atrophy (MSA), and dementia with Lewy bodies (DLB). Studies have shown that upwards of 80% of those with iRBD are likely to develop PD, DLB, or MSA within a span of about 16 years (2–4). These afflictions are characterized by their severely debilitating impact, poor prognoses, and substantial reduction in the quality of life and longevity of sufferers. Consequently, a deep understanding of the risk factors and underlying mechanisms of iRBD is crucial for effective management and potentially for the prevention of its progression to more grave neurodegenerative diseases.

Numerous studies have established a strong link between Type 2 Diabetes Mellitus (T2DM) and alpha-synucleinopathies. Observational studies identify T2DM as an independent risk factor for PD (5–9) and DLB (10, 11), exacerbating symptoms and accelerating disease progression. Furthermore, pancreatic tissue samples from both T2DM and α-synucleinopathy patients show similar pathological changes, notably the deposition of phosphorylated α-synuclein in pancreatic β cells, suggesting parallel pathological mechanisms in these diseases (12). *In vitro* experiments have demonstrated that the characteristic deposit protein in diabetes, islet amyloid polypeptide (IAPP), interacts with alpha-synuclein, promoting its aggregation (13). Animal studies further indicate that the hyperglycemic state and insulin resistance in diabetes may trigger immune-inflammatory responses, facilitating the accumulation of α-synuclein and the onset of neurodegenerative changes (14, 15). These findings collectively support the role of T2DM in promoting α-synucleinopathies.

However, research on the relationship between T2DM and the precursor stage of alpha-synucleinopathies, iRBD, remains scarce and controversial. Using linkage disequilibrium score regression (LDSC), Krohn’s team identified a genetic correlation between T2DM and iRBD (rg = 0.66, se = 0.23, p = 0.0047) (16). In a community-based observational study, Wong and colleagues pinpointed diabetes as an independent risk factor for people with probable RBD (OR[95%] =1.37[1.04-1.82]) (17), while research from José Haba’s team refuted this association (18). It is pivotal to recognize that observational studies are vulnerable to confounding bias and reverse causality.

Mendelian Randomization (MR) is a statistical tool employing genetic variants as instruments to deduce the causal impact of exposures on outcomes (19). This method leverages the fact that the allocation of genetic variants at conception is impervious to environmental or lifestyle influences, making MR estimates more resistant to confounding factors and reverse causation biases (20). Moreover, genetic variant data are typically derived from genome-wide association studies (GWAS), known for their large sample sizes, consequently enhancing MR’s capability to robustly detect causal relationships (21).

To reduce biases from confounders and reverse causality, and to provide robust genetic evidence on the relationship between T2DM and iRBD, we conducted a two-sample MR analysis, considering T2DM as the exposure and iRBD as the outcome. Given that T2DM can lead to a range of complications through immune-inflammatory responses (22, 23), and abnormalities in cytokine levels and immune cell characteristics are observed in iRBD patients (24–27), we conducted a network MR analysis. This analysis focused on immune-related circulating protein levels, inflammatory factors, and immune cell characteristics as potential mediators to investigate the underlying immune-inflammatory mechanisms by which T2DM may lead to iRBD.

## 2 Materials and methods

### 2.1 Study design

**Figure 1:**
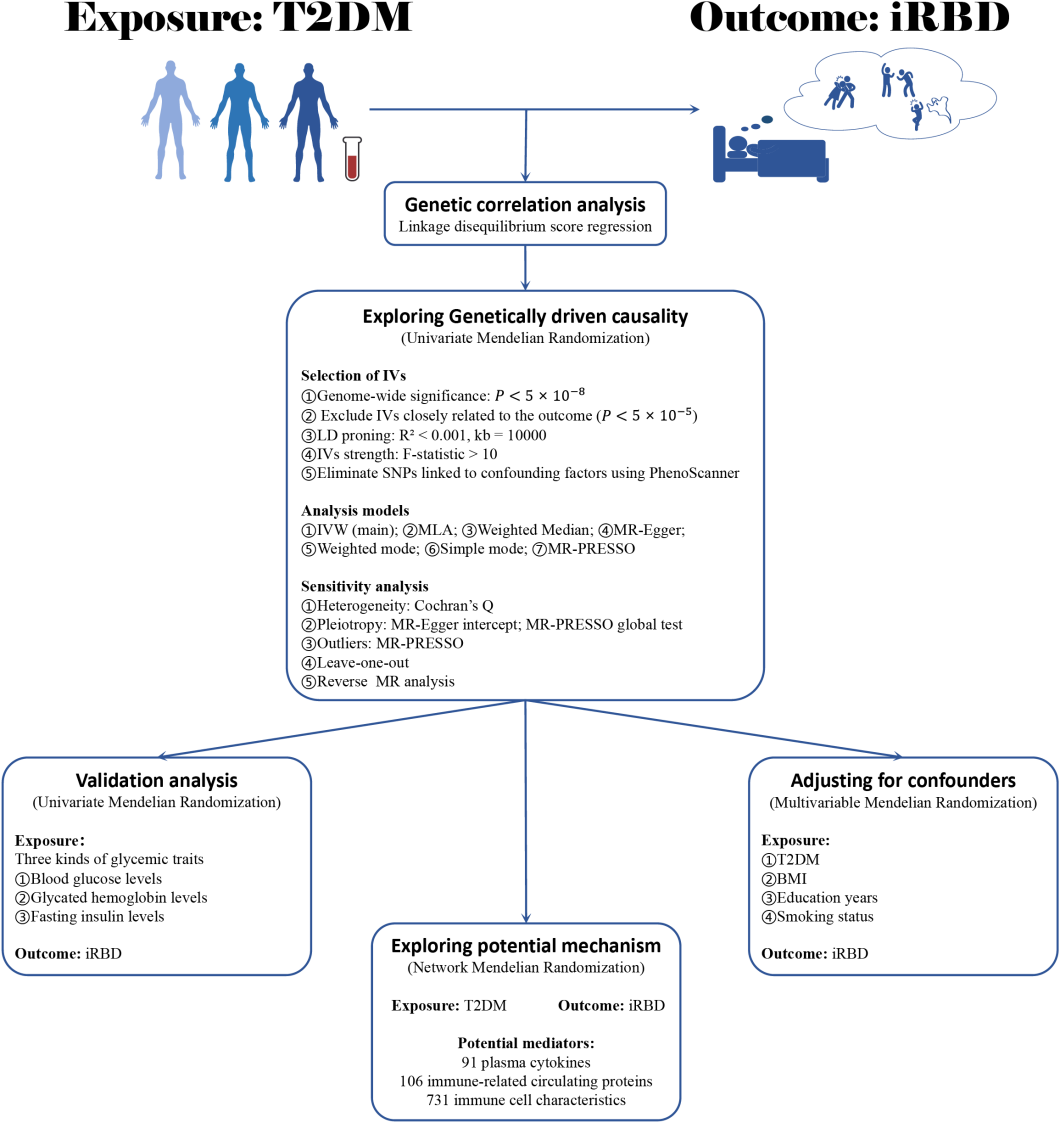
The flowchart of study design. BMI, body mass index; T2DM, type 2 diabetes mellitus; iRBD, isolated REM sleep behavior disorder; MR, Mendelian randomization; IV, instrumental variables; IVW, Inverse variance weighted; MLA, Maximum likelihood analysis.

**Figure 1** delineates the methodology of our two-sample MR analysis, exploring the link between T2DM and iRBD. Initially, we employed LDSC to assess the genetic correlation between these two conditions. Following this, with T2DM as the exposure factor and iRBD as the outcome factor, we conducted a univariate Mendelian Randomization analysis (UVMR) to investigate their gene-driven causal relationship. To further corroborate this result, UVMR was again utilized, to examine the connection between three glucose metabolic traits (blood glucose, glycated hemoglobin, and fasting insulin levels) and iRBD. The next phase of our study involved a multivariable MR (MVMR) method, integrating confounders like Body Mass Index (BMI), smoking habits, and educational level, to validate the robustness and consistency of the T2DM-iRBD association. Lastly, our analysis extended to a network MR approach with cytokines, immune-related circulating protein levels, and immune cell characteristics as potential mediators, delving into the underlying mechanisms of T2DM leading to iRBD. Our methodology adheres rigorously to the STROBE-MR guidelines (28).

### 2.2 Data sources

To strengthen causality inference and reduce bias from small sample sizes, we carefully selected the most comprehensive GWAS datasets for T2DM and iRBD available from the GWAS Catalogue (https://www.ebi.ac.uk/gwas/), focusing on individuals of European descent. The T2DM GWAS dataset, specifically, was derived from a meta-analysis conducted by Angli Xue’s team, combining data from MAGIC, DIAGRAM, and GERA, encompassing a total of 659,316 participants (29). For the outcome data, we chose a GWAS dataset of an iRBD cohort (1,061 cases and 8,386 controls) compiled by the International RBD Study Group (16). The iRBD cases were diagnosed using the International Classification of Sleep Disorders (2nd or 3rd Edition), inclusive of video polysomnography assessments. To ensure better comparability between the case and control groups, principal components analysis was used to adjust for population substructure, accounting for variables like gender and age. Data concerning metabolic traits related to blood sugar, confounders (BMI, education years, smoking status) and potential mediators (91 plasma cytokines, 106 immune-related circulating proteins, 731 immune cell characteristics) were sourced from the IEU online database (https://gwas.mrcieu.ac.uk/) and the GWAS Catalogue database (**Table 1**). All the aforementioned GWAS data are publicly accessible. Importantly, we retrieved exposure and outcome data from independent samples to avoid bias stemming from sample overlap. 

**Table 1:** Description of the data source of the study.

Trait	Data source	Sample size	Description
**Exposure**			
Type 2 diabetes	GWAS Catalog ID: GCST006867	659316	DIAGRAM: 34840 cases and 114981 controls; GERA: 6905 cases and 46983 controls; UKB: 21147 cases and 434460 controls
	PMID: 30054458		
Blood glucose levels	IEU ID: ebi-a-GCST90014005	357580	Data from the UK Biobank
	PMID: 34017140		
HbA1c levels	IEU ID: ebi-a-GCST90014006	389889	
	PMID: 34017140		
Fasting insulin levels	IEU ID: ebi-a-GCST90002238	151013	
	PMID: 34059833		
**Confounder**			
BMI	IEU ID: ukb-b-19953	461460	
Education years	IEU ID: ebi-a-GCST90029013	461457	
	PMID: 29892013		
Smoking status	IEU ID: ebi-a-GCST90029014	468170	
	PMID: 29892013		
**Mediator**			
91 plasma cytokines	GWAS Catalog ID: GCST90274758 to GCST90274848	14824	Data from 11 independent cohorts
	PMID: 37563310		
106 immune-related circulating proteins	IEU ID: ebi-a-GCST90019380 to ebi-a-GCST90019485	10708	Data from the Fenland study
	PMID: 33328453		
731 immune cell characteristics	IEU ID: ebi-a-GCST90001391 to ebi-a-GCST90002121	3757	Data from the SardiNIA dataset
	PMID: 32929287		
			
**Outcome**			
iRBD	GWAS Catalog ID: GCST90204200	9447	The iRBD cohort (N cases = 1061, N controls = 8386) included large cohorts of French, French, Canadian, Italian and British origins, and smaller cohorts from different European populations. IRBD cases were diagnosed according to the International Classification of Sleep Disorders (2nd or 3rd Edition), including video polysomnography.
	PMID: 36470867		

MAGIC, the Meta-Analyses of Glucose and Insulin-related traits Consortium; DIAGRAM, diabetes Genetics Replication and Meta-analysis; GERA, genetic Epidemiology Research on Adult Health and Aging; IEU, Integrative Epidemiology Unit; The SardiNIA project, a long-term research initiative involving participants who are natives of the central eastern coastline of Sardinia, Italy.

### 2.3 Genetic correlation analysis

In our study, we employed single-variate LDSC to estimate the heritability of both T2DM and iRBD. Additionally, to clarify the genetic correlations between these two conditions, we utilized the bivariate LDSC method. (30–34).

### 2.4 Instrumental variable selection

Genetic variants were selected as instrumental variables based on strong associations with the exposure (*P* < 5 × 10^-8^). To mitigate reverse causation bias, we excluded SNPs highly associated with the outcome ( $P\;<\;5\times 10^{-5}$ ) (35). To quantify the proportion of variance in exposure, the $R^{2}\;$ clue of single nucleotide polymorphisms (SNPs) was estimated by effect estimates (beta) and allele frequencies (EAF): $R^{2}=\;2\;\times \;EAF\;\times \;\left(1\;-\;EAF\right)\times \;beta^{2}$ (36). The instrument strength was estimated using the F-statistic: $F\;=\frac{R^{2}}{\left(1-R^{2}\right)}\times \frac{\left(N\;-\;k\;-\;1\right)}{k}$ , where N represents the sample size and k is the number of instrumental variables (37). Only the SNP of F-statistic > 10 could be included in this study. A minor allele frequency (MAF) ≥0.01. We clumped independent SNPs based on European ancestry reference data (1000 Genomes Project, r²>0.001, genomic region=10,000 kb). Summary statistics were harmonized on alleles positively associated with exposures. Ambiguous palindromic SNPs (A/T, C/G) with a MAF>0.3 were excluded (38). To fulfill the independence assumption for MR analysis, SNPs associated with various factors, including BMI, smoking, alcohol consumption, years of schooling, occupation, head injury history, olfactory dysfunction, antidepressant drug usage, and antipsychotics usage (16–18, 39–41), were excluded based on the PhenoScanner query results. SNPs associated with confounding factors were displayed in **Supplementary Table S4**. The statistical power of the Mendelian randomization analysis was computed using the tool available at https://shiny.cnsgenomics.com/mRnd/ (42).

### 2.5 Statistical analysis

The primary analysis utilized the inverse-variance weighted (IVW) method (43), benchmarked against other MR methods robust to pleiotropy, including the maximum likelihood method (44), weighted median (45), weighted mode (46), MR-Egger regression (47) simple mode (48) and the MR-PRESSO (49). We initially investigated the genetically driven causal relationship between T2DM as the exposure and iRBD as the outcome. Subsequently, to validate our findings, we delved into the causal connections between three key blood glucose metabolic traits—blood glucose levels, glycated hemoglobin levels, and fasting insulin levels—and iRBD. Although MR effectively reduces confounding bias, it does not completely eliminate its influence. If the instrumental variables used in the analysis are associated with known risk or protective factors for the outcome, they violate the second assumption of MR, leading to biased results. Therefore, we employed MVMR to adjust for instrumental variables closely related to BMI, years of education, and smoking habits (16–18, 39–41), in order to assess the robustness of the relationship between T2DM and iRBD.

### 2.6 Network Mendelian randomization analyses

To investigate the potential immune-inflammatory mechanisms by which T2DM affects iRBD, we selected 91 plasma cytokines, 106 immune-related circulating proteins, and 731 immune cell characteristics as candidate mediators and conducted a network MR analysis—an MR-based mediation analysis. We sequentially calculated the total effect of T2DM on iRBD, the direct effect of T2DM on the mediators, and the direct effect of the mediators on iRBD. The magnitude of the mediation effect was estimated using the product-of-coefficients method, and the confidence interval was determined using the Delta method (50).

### 2.7 Sensitivity analyses

Cochran’s Q statistic assessed heterogeneity, serving as a potential indicator of pleiotropy in IVW estimators. Horizontal pleiotropy was identified through the p-value for the intercept in MR-Egger (47) and the global test in MR-PRESSO (49). MR-PRESSO also pinpointed outliers, prompting a reanalysis of all MR methods after outlier SNP exclusion. Leave-one-out analyses determined if a single SNP unduly influenced estimates.

All statistical tests were two-sided, a P value of less than 0.05 was considered statistically significant. All above statistical analyses were conducted by using the R-statistical software (version 4.3.1) with related R packages.

## 3 Results

### 3.1 Characteristics of the selected instrumental variables

**Supplementary Table S2** details the selected genetic instruments for our MR analyses. These genetic variants were strongly associated with their respective exposures, explaining a variance that ranged from 0.26% to 21.81%. Importantly, all instrumental variables had F-statistics exceeding 10, indicating that our results are unlikely to be affected by weak instrument bias. In terms of statistical power within our MR analysis, except for the exposure factor of fasting insulin with a power value of 0.86, all other exposures had power values of 1, signifying robust statistical power to detect potential causal relationships.

### 3.2 Gene-driven causal relationship between T2DM and iRBD

**Figure 2:**
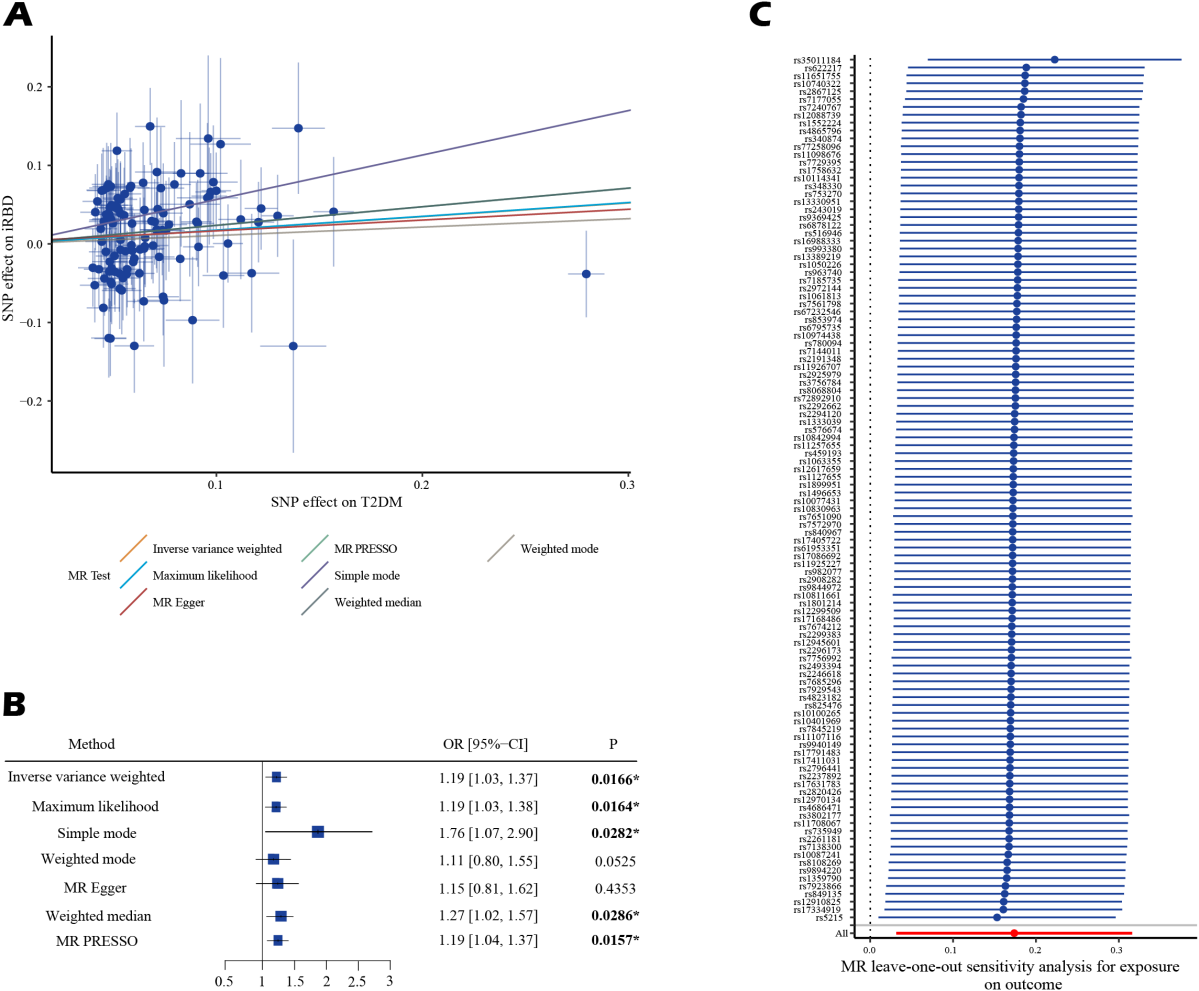
The estimated causal effects of T2DM on iRBD using MR analysis. **(A)** presents the results using a scatter plot. **(B)** presents the results using a forest plot. **(C)** presents the results of leave-one-out analysis. Effect sizes are represented as odds ratios (OR), and the horizontal bars represent 95% confidence intervals (CIs). Statistically significant P-values are indicated with superscript letters and asterisks (*).

Bivariable LDSC analysis indicated a genetic correlation between T2DM and iRBD (rg = 0.306, P = 0.029;, **Table 2**; **Supplementary Table S1**). The UVMR analysis corroborated a gene-driven causal relationship, with an odds ratio (OR) of 1.19 (95% CI: 1.03-1.37; P = 0.017, **Figures 2A, B**). Subsequent secondary analyses using various methods supported these findings: maximum likelihood yielded an OR of 1.19 (95% CI: 1.03, 1.38, P = 0.016), weighted median produced an OR of 1.27 (95% CI: 1.02, 1.57, P = 0.031), simple mode estimated an OR of 1.76 (95% CI: 1.05, 2.96, P = 0.035), and MR-PRESSO calculated an OR of 1.19 (95% CI: 1.04, 1.27, P = 0.016). Notably, all analyses excluded the instrumental variable ‘rs13239186’ due to its strong association with smoking as identified in the PhenoScanner database, to avoid confounding bias (**Supplementary Tables S2**, **4**). Sensitivity analyses, using Cochran’s Q statistic, showed no heterogeneity in the MR results. Furthermore, MR-Egger’s intercept and MR-PRESSO’s global test indicated no evidence of horizontal pleiotropy. MR-PRESSO also detected no outlier effects (**Supplementary Table S5**). Leave-one-out analysis suggested that the results were not driven by any single SNP (**Figure 2C**; **Supplementary Table S6**). Reverse Mendelian randomization did not support the existence of reverse causation (**Supplementary Table S7**).

**Table 2:** Results of LDSC between T2DM and iRBD.

Exposure	Outcome	rg	se	P-value
T2DM	iRBD	0.31	0.14	0.029*

LDSC, Linkage disequilibrium score regression; rg, genetic correlation; se, standard error. Results that achieve statistical significance will be highlighted in bold and marked with an asterisk (*).

In our UVMR analysis examining the relationship between three blood sugar metabolism factors and iRBD, we discovered that elevated blood glucose levels increase the risk of iRBD, with an OR of 1.55 (95% CI: 1.04, 2.30, P = 0.03). Secondary analyses using Maximum likelihood and MR-PRESSO corroborated these findings, with both methods indicating an OR of 1.55 (95% CI: 1.04, 2.31, P = 0.03 for Maximum likelihood; 1.55, 95% CI: 1.04, 2.30, P = 0.03 for MR-PRESSO). Significantly, our analyses systematically excluded the instrumental variables ‘rs8047587’ and ‘rs34402524’, due to their strong associations with alcohol consumption and years of education, respectively (**Supplementary Table S4**). There was no evidence to suggest a causal link between HbA1c or fasting insulin levels and iRBD. Sensitivity analysis indicated heterogeneity in the relationship between HbA1c levels and iRBD (Q-value=0.01), and potential horizontal pleiotropy (Global P-value =0.01). The remaining analyses showed no disturbances from heterogeneity, horizontal pleiotropy, or outliers (**Table 3**; **Supplementary Tables S8**, **9**).

**Table 3:** Causal relationships between glucose metabolic traits and iRBD performed by MR.

Method	nSNPs	OR (95%)	P-value	Q-value	Intercept P-value	Global P-value
**Blood glucose levels**						
Inverse variance weighted	94	1.55 (1.04, 2.30)	0.03*	0.45		
Maximum likelihood	94	1.55 (1.04, 2.31)	0.03*			
MR Egger	94	1.69 (0.83, 3.42)	0.15		0.77	
Weighted median	94	1.45 (0.79, 2.65)	0.23			
MR PRESSO	94	1.55 (1.04, 2.30)	0.03*			0.48
**HbA1c levels**						
Inverse variance weighted	299	1.12 (0.85, 1.47)	0.43	0.01*		
Maximum likelihood	299	1.12 (0.88, 1.42)	0.37			
MR Egger	299	0.90 (0.55, 1.47)	0.68		0.3	
Weighted median	299	1.16 (0.78, 1.71)	0.47			
MR PRESSO	299	1.12 (0.85, 1.47)	0.43			0.01*
**Fasting insulin levels**						
Inverse variance weighted	22	0.51 (0.14, 1.91)	0.32	0.86		
Maximum likelihood	22	0.52 (0.14, 1.95)	0.33			
MR Egger	22	0.09 (0.00, 9.26)	0.32		0.44	
Weighted median	22	0.76 (0.12, 4.68)	0.77			
MR PRESSO	22	0.51 (0.17, 1.52)	0.24			0.87

nSNPs, the number of Single Nucleotide Polymorphisms; Q-value, Cochran’s Q statistic P value; Intercept P-value, MR Egger intercept P value; Global P-value, MR PRESSO global test P value. Results that achieve statistical significance will be highlighted in bold and marked with an asterisk (*).

Study conclusions were supported by weighted-median estimation, weighted-mode, and MR-Egger methods (**Supplementary Table S2**). Cochran’s Q statistic indicated no significant heterogeneity in SNP effects (p > 0.05). No evidence of potential horizontal pleiotropy was detected for the eight factors identified (p > 0. 05). To further assess the robustness of the results, we conducted MR-PRESSO tests on the included SNP loci. In addition, a leave-one-out sensitivity analysis was conducted to assess the influence of each SNP on the overall causal relationship. The results demonstrated that systematically removing individual SNPs and repeating the MR analysis did not reveal significant differences in the observed causal relationships (**Supplementary Tables S3**–**5**).

### 3.3 Results for multivariable MR for adjusting confounders

**Figure 3:**
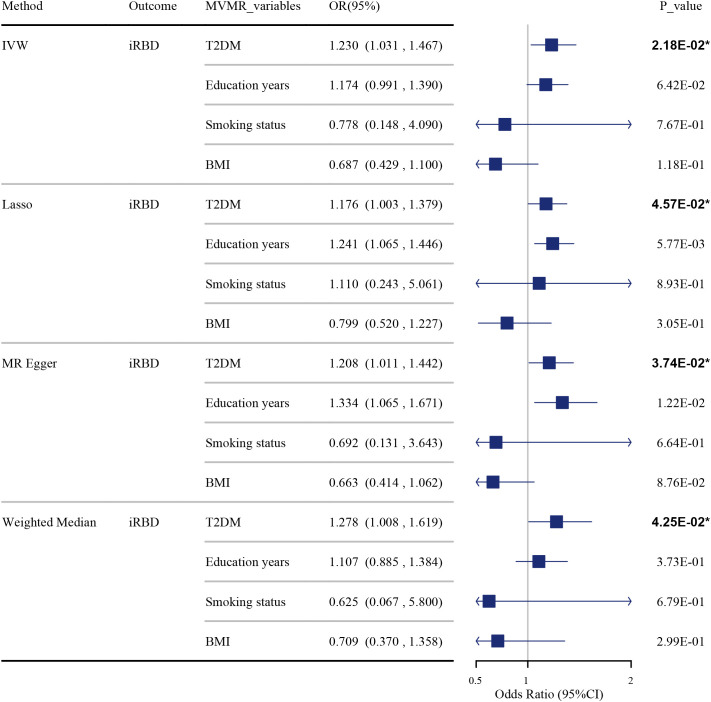
The impact of T2DM on iRBD in the MVMR analysis after adjusting for confounders. Effect sizes are represented as odds ratios (OR), and the horizontal bars represent 95% confidence intervals (CIs). Statistically significant P-values are indicated with superscript letters and asterisks (*).

The results of the MVMR suggest that T2DM remains an independent risk factor for iRBD, even after adjusting for potential confounders such as years of education, smoking status, and BMI. This was demonstrated through various analytical methods: IVW-OR at 1.230 (95% CI: 1.031, 1.467, P = 0.022), Lasso-OR at 1.176 (95% CI: 1.003, 1.379, P = 0.046), MR-Egger-OR at 1.208 (95% CI: 1.011, 1.442, P = 0.037), and Weighted Median-OR at 1.278 (95% CI: 1.008, 1.619, P = 0.043) as shown in **Figure 3** and **Supplementary Table S10**. Sensitivity analysis revealed no horizontal pleiotropy in the multivariable MR analyses, as indicated by MR-Egger regression (MR Egger intercept P value = 0.092).

### 3.4 Network MR analyses to explore potential mechanisms

In our network MR analysis (**Table 4**), we identified significant associations: T2DM was observed to decrease plasma levels of Stromal Cell-Derived Factor 2 (SDF-2) (P=8.03E-03), with lower SDF-2 levels elevating the risk of iRBD (P=1.75E-03). The mediation effect of SDF-2 in the T2DM-iRBD relationship accounted for 7.03% (95% CI 0.11%–7.70%). Furthermore, T2DM was associated with an increase in BAFF-receptor (BAFF-R) on IgD- CD38- B cells (P = 3.08E-03), a characteristic that also heightened the risk of iRBD (P = 7.43E-04). The mediation effect of this immune characteristic in the T2DM-iRBD causal pathway was 11.17% (95% CI 6.22%–11.65%). Notably, none of the network MR analyses indicated the presence of heterogeneity and horizontal pleiotropy (**Supplementary Tables S11**, **12**).

**Table 4:** Mediation effect of immune and inflammatory factors in the association between T2DM and iRBD.

Exposure	Mediator	Outcome	Direct effect α			Direct effect β			Mediation effect	
			Beta	SE	P-value	Beta	SE	P-value	Effect size (95%CI)	Proportion % (95%CI)
T2DM	①SDF-2 levels	iRBD	-0.056	0.021	8.03E-03	-0.217	0.069	1.75E-03	1.21E-02(3.20E-05,2.42E-02)	7.03(0.11,7.70)
	②BAFF-R on IgD- CD38- B cell		0.113	0.038	3.08E-03	0.17	0.05	7.43E-04	1.92E-02(1.87E-03,3.66E-02)	11.17(6.22,11.65)

SDF-2, Stromal Cell-Derived Factor 2. BAFF-R, BAFF-receptor.

Direct effect α: The causal effect of T2DM on mediators in two sample-MR analysis.

Direct effect β: The causal effect of mediators on iRBD in two sample-MR analysis.

Mediation effect: The effect of T2DM on iRBD mediated through mediators.

## 4 Discussion

In this pioneering MR study, we identified a significant association between elevated blood glucose levels, T2DM, and increased risk of iRBD. This link remained strong even after adjusting for BMI, smoking, and education. Mediation analysis highlighted an indirect pathway where T2DM contributes to iRBD by increasing BAFF-R on IgD- CD38- B cells and reducing plasma SDF-2 levels. These findings offer valuable insights into screening high-risk populations for iRBD and understanding its pathogenesis, representing a notable advance in the field.

Numerous observational studies have consistently demonstrated that T2DM is an independent risk factor for alpha-synucleinopathies such as PD (5–9) and DLB (10, 11), contributing to increased incidence, exacerbated symptoms, and accelerated disease progression. Valbuena’s team, through examination of pancreatic tissue slides, identified similar pathological changes in pancreatic β cells of both T2DM and alpha-synucleinopathy patients, notably the presence of phosphorylated alpha-synuclein (12). More significantly, Horvath’s group discovered *in vitro* that islet amyloid polypeptide (IAPP), a primary pathogenic deposit in T2DM patients, promotes the accumulation of alpha-synuclein amyloid (13). Furthermore, basic experimental findings indicate that insulin resistance and hyperglycemia can trigger immune-inflammatory responses, thereby fostering the deposition of alpha-synuclein and the onset of neurodegenerative changes (14, 15). These studies collectively highlight a profound connection between T2DM and alpha-synucleinopathies.

Research on the link between T2DM and iRBD, a precursor to alpha-synucleinopathies, is limited and presents conflicting findings. Wong et al. found diabetes to be an independent risk factor for probable RBD in a community-based observational study (17). In contrast, a Swiss study reported no such association (18). It’s crucial to acknowledge that the conclusions of observational studies may be skewed by confounding factors and reverse causality. Our research identified a genome-wide genetic correlation between T2DM and iRBD. Furthermore, we found that T2DM increases the incidence of iRBD, a conclusion supported by multiple models and robust against heterogeneity, pleiotropy, and outliers. The absence of a similar conclusion in the Swiss observational study may be attributed to its limited statistical power due to a small sample size, encompassing only 24 iRBD cases.

The exact mechanisms by which T2DM fosters the development of iRBD remain elusive. Previous research has demonstrated that high blood glucose can induce PD by modulating oxidative stress, and triggering inflammation reaction (51–57). Yi-Qing Lv and his team found that hyperglycemia leads to severe neuroinflammation and accelerates α-synuclein deposition in the central nervous system of mice (15). Similarly, Yan Sun and colleagues found a close link between fasting plasma glucose levels and the increased accumulation and phosphorylation of α-synuclein in the cortex, pre-commissural putamen, and dopaminergic neurons in the substantia nigra of T2DM monkeys’ brains (58). Based on these findings, we speculate that in T2DM patients, hyperglycemia-induced neuroinflammation and α-synuclein deposition may affect brain regions that inhibit spinal motor neurons during REM sleep (59), potentially triggering iRBD (59).

Our network MR analysis reveals that T2DM may promote the onset of iRBD by increasing BAFF-R on IgD- CD38- B cells and reducing circulating SDF-2 levels. Previous research has shown that T2DM, through hyperglycemia and insulin resistance, elevates pro-inflammatory cytokines and chemokines, triggering abnormal immune cell activation and tissue infiltration, leading to various complications (22, 23). Similar disturbances in cytokine levels and immune cell characteristics have been observed in iRBD patients (24–27). SDF-2, a small protein located in the endoplasmic reticulum, is closely linked with endoplasmic reticulum stress regulation (60). While its precise role remains unclear, low tissue expression of SDF-2 is associated with poor prognosis in various tumors. Additionally, SDF-2 can enhance nitric oxide (NO) release by interacting with endothelial nitric oxide synthase (eNOS), reducing the incidence of several cardiovascular diseases (61). Consequently, SDF-2 may have a protective role in the onset and progression of various diseases, including iRBD. The specific mechanisms by which T2DM regulates SDF-2 and its impact on iRBD, however, require further investigation. BAFFR, encoded by the TNFRSF13C gene and serving as a critical pro-survival receptor in cells (62, 63), is notably upregulated in T2DM within IgD- CD38- B cells. This upregulation boosts both the survival and proliferation of these cells, implying a potential role in iRBD development through an unidentified toxic immune response. Elucidating the specific mechanisms of this process demands more comprehensive research.

In summary, our study reveals the causal relationship between T2DM and iRBD, as well as the underlying immune-inflammatory mechanisms. This finding offers new insights for screening high-risk populations for iRBD and has significant public health implications. It is essential to regularly screen T2DM patients for sleep disorders, particularly when they frequently experience abnormal dreams or unusual behaviors during sleep, as this may indicate the comorbidity of T2DM and iRBD. More importantly, future research should explore whether T2DM-targeted treatments, such as hypoglycemic agents, insulin preparations, and GLP-1 receptor agonists, could serve as potential treatments for iRBD. Additionally, it is worth investigating whether interventions targeting specific inflammatory proteins and immune cells in diabetic patients can reduce the risk of iRBD.

Our study has several limitations. First, although we employed various analytical methods to validate our findings, larger iRBD GWAS sample sizes are still needed for MR analysis to further confirm the reliability of the results. A larger sample size would not only improve the precision of genotype-phenotype association estimates in GWAS data but also help reduce selection bias caused by specific population characteristics. Second, all GWAS data used in this study were derived from European populations, which limits the generalizability of our conclusions to other ethnic groups. Future research should include cross-ethnic MR analyses to further assess the robustness of our findings. Third, as our conclusions are primarily based on MR analysis, and previous cross-sectional studies on the relationship between T2DM and iRBD have shown inconsistent results, multi-center cohort studies are needed to provide real-world evidence supporting these conclusions. Fourth, it is important to acknowledge that MR analysis reflects the relationship between genetically driven exposures and outcomes, but genetic factors only account for part of the exposure, highlighting the inherent limitations of MR analysis. Finally, while this study has preliminarily identified the potential immune-inflammatory mechanisms by which T2DM may lead to iRBD, we have not explored the cell-cell interactions and related signaling pathways in depth. With the development of advanced analytical platforms (64–66), future research should focus on further mechanistic insights.

## 5 Conclusions

Our study indicates that T2DM and hyperglycemic conditions are risk factors for iRBD. Additionally, T2DM may indirectly contribute to the onset of iRBD by upregulating BAFFR expression in IgD- CD38- B cells and reducing circulating SDF-2 levels. Our findings offer new directions for screening high-risk populations for iRBD. The comorbidity of T2DM and iRBD, as well as their potential immune-inflammatory mechanisms, warrant further investigation in future studies.

## Data Availability

The original contributions presented in the study are included in the article/**Supplementary Material**. Further inquiries can be directed to the corresponding authors.
